# Using Doppler sonography resistive index for the diagnosis of perinatal asphyxia: a multi-centered study

**DOI:** 10.1186/s12883-022-02624-2

**Published:** 2022-03-19

**Authors:** Parisa Pishdad, Fatemeh Yarmahmoodi, Tannaz Eghbali, Peyman Arasteh, Seyyed Mostajab Razavi

**Affiliations:** 1 Medical Imaging Research Center, Department of Radiology, School of Medicine, Shiraz University of Medical Sciences, Shiraz, Iran; 2 Shiraz University of Medical Sciences, Shiraz, Iran; 3 Department of Pediatrics, School of Medicine, Neonatal Research Center, Shiraz University of Medical Sciences, Shiraz, Iran

**Keywords:** Asphyxia, Perinatal, Ultrasonography, Doppler, Magnetic resonance imaging, Resistive index

## Abstract

**Background and objective:**

Inhere we evaluated the diagnostic utility of Doppler sonography (DS) of the anterior cerebral artery (ACA), middle cerebral artery (MCA) and the basilar arteries (BA) based on resistive index (RI) for the diagnosis of asphyxia.

**Methods:**

In this multi-centered cross-sectional study, neonates with clinical diagnosis of asphyxia, were considered for study. During the first 24 h, neonates underwent DS. MRI was done for each neonate during the first month, after discharge or during hospital admission, after obtaining clinical stability. Staging based on DS was compared with staging based on MRI.

**Results:**

Overall, 34 patients entered the study. DS of the ACA, MCA, BA all had significant correlation with MRI findings (regarding severity of asphyxia) (*r* > 0.8 and *p* < 0.001).

In the receiver-operating-characteristic analysis, ideal cut-off point for diagnoses of asphyxia based on ACA and BA was RI ≤ 0.62 [area under the curve (AUC) = 0.957 and 95% CI: 0.819–0.997; sensitivity = 95.65; specificity = 100; positive predictive value (PPV) = 100; negative predictive value (NPV) = 90.9 and negative likelihood ratio (NLR) = 0.043]. Regarding MCA, similarly, a RI ≤ 0.62 was ideal for differentiating between normal and asphyxiated neonates (AUC = 0.990 and 95% CI: 0.873–1; sensitivity = 91.30; specificity = 100; PPV = 91.2; NPV = 100 and NLR = 0.087).

**Conclusion:**

For evaluating neonates clinically suspected of asphyxia, especially in centers with limited facilities such as MRI, DS can be used as a first line diagnostic modality and RI of ≤ 0.62 is an appropriate cut-off for the diagnosis of perinatal asphyxia.

## Background

Multiple events during labor and delivery may lead to encephalopathy of the neonate which is generally termed perinatal asphyxia [1]. Asphyxia is one of the most common causes of mortality and morbidity among newborns and an estimated one to six from every one thousand full-term births are associated with some degrees of asphyxia [2].

The events that lead to asphyxia cause oxygen deprivation and finally may present as neurological deficit in the neonate. Asphyxia may present with different symptoms including abnormal level of consciousness, difficult respiration, decreased muscle tone and reflexes and etc. [3].

As asphyxia prolongs, cerebral blood flow is compromised and decreases through systemic hypotension and dysregulation within the cerebral regulatory system. This leads to hypoperfusion and ischemia, which may develop into hypoxic-ischemic encephalopathy and neurological impairment (blindness, hearing loss, cerebral palsy, motor and mental development problems) for the child [1, 4, 5].

Different modalities have been used to diagnose asphyxia among neonates, among which magnetic resonance imaging (MRI) is considered the gold standard imaging method used in clinical practice [6]. Other imaging modalities include ultrasonography, Doppler sonography (DS) of the cerebral arteries, and in some cases computed tomography (CT) [7].

Sonography based imaging, present a non-invasive, portable and safe method of evaluation for patients suspected of asphyxia [8]. A reduction in the Resistive index and an increase in the end-diastolic flow velocity have been associated with changes in cerebral blood flow and presents an effective method for evaluation of asphyxia [9].

In a recent study by Kudreviciene et al., children with asphyxia were studied using DS. They determined a cut-off based on DS index for the prediction of neuro-developmental injuries. Other studied have also studied the prognostic role of DS in children with ischemic brain damage [4, 10, 11], however at present, data on the diagnostic role of DS in asphyxia remains scarce and DS is not routinely used in the clinical assessment of asphyxia in neonates [9].

In this study we evaluated and compared DS and MRI findings in a sample of neonates suspected of asphyxia, we also determined an optimum cut-off point based on DS (index) of the cerebral arteries to differentiate between neonates with asphyxia and normal neonates.

## Methods

### Study design

This is a multi-centered cross-sectional study conducted in Namazi, Hafez and Hazrat Zeinab hospitals, affiliated to Shiraz University of Medical Sciences, Shiraz, Iran. During January 2016 to July 2016, neonates who were admitted with a diagnosis of asphyxia based on the Sarnat and Sarnat clinical criteria [12] in the mentioned health care centers, were considered for entry in the study. Neonates born between 37 and 42 weeks who had signs of fetal distress such as abnormal fetal monitoring and presence of meconium in the amnion, with an umbilical cord blood PH of less than 7.1, five minute Apgar score of less than five, were included in the study.

Neonates born earlier than 37 weeks or later than 42 weeks, those who had any congenital disorders (for example patent ductus arteriosus), and those with myocardial dysfunction, were excluded from the study.

We excluded patients with myocardial dysfunction and congenital abnormalities as some studies have shown these patients to have variable findings regarding DS indexes [13].

### Protocol and patient evaluation

Patients were initially evaluated by a neonatologist and were scored based on the Sarnat and Sarnat scoring system (previously mentioned) as mild, moderate and severe asphyxia. After diagnosis of asphyxia through clinical evaluation by the neonatologist, and after patients were clinically stable (normal vital signs and normal blood gas indices) and had normal hematocrit, during 6 to 24 h after birth, each patient underwent DS (Shenzhen Mindray Bio-Medical Electronics Co., China) of the cerebral arteries using a curved probe of 5 MHz and a linear probe of 7.5 MHz. As DS studies are dynamic, DS was done after the first 6 h (up to 24 h) of birth in order to obtain stability and more importantly to minimize changes in DS findings. Moreover, none of our patients received hypothermia treatment in our center.

For DS of the ACA, imaging was conducted on bilateral sides on parasagittal planes through the anterior fontanelles. Parameters related to the ACA were measured using a branch of the anterior artery, which were anterior to the corpus callosum. For the MCA, imaging was conducted on bilateral sides by both temporal bones between the eye socket and the ear above the zygoma on axial planes. For the BA, imaging was obtained in the sagittal planes located just before the pons. Examination of the neonates was performed during sleep.

Magnetic resonance imaging (MRI) was done for each neonate during the first month (a median of 12 days), after discharge or during hospital admission, when the patients obtained a clinically stable condition. Scoring of MRI findings was done based on the system introduced by Weeke et al. [14]. Accordingly, limited hyper intense T2 signal in the brain cortex and subcortical white matter represented mild degree of hypoxic ischemic encephalopathy. Involvement of the basal ganglia and thalamus was considered severe hypoxic ischemic encephalopathy. Moreover, mild to moderate encephalopathy were considered disseminated signal changes of the cortex and subcortical weight matter [15]. Accordingly, patients were categorized into three groups of normal, mild to moderate (stage 2) and severe (stage 3) regarding asphyxia.

MRI and sonography findings were interpreted by two different radiologists who were both unaware of the clinical staging and the staging done by the other radiologist.

Data including sex, one minute and five minute APGAR scores, type of delivery, birth weight, gestational age, clinical severity of asphyxia, severity of asphyxia based on sonography (based on ACA, MCA and BA), and severity of asphyxia based on MRI findings, were all registered in a data gathering sheet.

We used the Sarnat and Sarnat criteria for clinical diagnosis and classification of severity of asphyxia. This criteria considers six variables for the diagnosis of asphyxia including: alertness, muscle tone, seizure, pupils, respiration pattern, and duration of symptoms [12].

For sonography-based staging of asphyxia, the Pourcelots's resistive index (RI) was used to estimate cerebral blood flow status, due to its easiness of use, reproducibility and its independence of the angle of insonation [16].

Peak systolic velocity (PSV), end diastolic velocity (EDV) and resistive index (RI) of the anterior cerebral arteries (ACA), middle cerebral artery (MCA), and the basilar arteries (BA) were measured and the severity of disease was defined, accordingly. Severity of disease was measured according to the RI, which is calculated as the peak systolic velocity minus the end diastolic velocity divided by the systolic velocity. The RI was measured three consecutive times for each artery and the average was considered the final RI.

RI provides a tool to evaluate the dynamics of cerebral blood flow and cerebral pressure [17]. Appropriate cut-off points for classifying patients based on RI as severe, moderate and mild were obtained using previous literature [18]. According to the mentioned study a cut-off of RI ≤ 0.57 was defined as severe asphyxia, moreover according to our final results for the diagnosis of asphyxia (using our own obtained cut-off point for the diagnosis of asphyxia based on RI) the rest of the cut-offs were categorized accordingly. According to our results and that of previous literature (as mentioned before), severe asphyxia was considered as RI ≤ 0.57, moderate as RI = 0.58–0.62, mild was considered as RI = 0.63–0.67, and normal DS was considered as RI = 0.68–0.72.

In the end, staging based on sonography was compared with the staging based on MRI findings.

All patients were hospitalized in the neonatal intensive care units and received related intensive care according to standard protocols, therefor factors such as thermoregulation and vasogenic edema which may have affected RI were controlled and were similar for all the patients.

### Sample size calculation

In order to determine a cut-off point for DS, considering a sensitivity of 99% and a specificity of 67% and an accuracy of 99% for a positive likelihood ratio of more than 1.8, a sample size of 30 individuals was required.

### Statistical analysis

Data was analyzed using the Statistical Package for Social Sciences software (SPSS Inc., Chicago, IL, USA) for windows, version 16.

In order to evaluate the linear correlation between DS findings of the ACA, MCA and BA with MRI, the Spearman's correlation test was used.

To determine the ideal cut-off point regarding RI of the three arteries, in discriminating between normal neonates and those with asphyxia (considering MRI as the gold standard), the receiver operating characteristics (ROC) analysis was used, reporting its area under the curve (AUC), sensitivity, specificity, positive predictive value (PPV), negative predictive value (NPV), positive likelihood ratio (PLR) and negative likelihood ratio (NLR), where appropriate. In addition, a pairwise comparison of ROC curves was performed between the ACA, MCA and BA to determine the difference between the examined arteries.

We calculated the optimum RI cut-off point using the Youden index [19]. Using this model, the ideal cut-off point on the ROC curve is considered optimum which has the maximum sensitivity + specificity. Upper and lower limits of cut-off points were also determined considering the point on the ROC curve with the highest sensitivity and highest specificity.

Data are presented as means ± standard deviations (SD) or frequency and percentage, where appropriate.

## Results

Overall, 34 patients entered the study. One patient died before having MRI and was excluded from the study. Patients' baseline characteristics are shown in Table 1.

**Table 1:** Patients' baseline characteristics^a^

Variables	Statistics
Sex—no. (%)	
Male	19 (58)
Female	14 (42)
Gestational age—wks	
37	10 (30.3)
38	12 (36.4)
39	10 (30.3)
40	1 (3)
Birth weight—gr	
< 2500	2 (6.1)
2500–4000	30 (90.9)
> 4000	0
One minute Apgar score	
≤ 5	26 (78.8)
> 5	7 (21.2)
Five minute Apgar score	
≤ 5	33 (100)
> 5	0
Birth type	
Normal vaginal delivery	17 (51.5)
Cesarean section	16 (48.5)
Clinical severity	
Stage 1	7
Stage 2	16
Stage 3	10
Severity based DS^b^	
Normal	7
Mild	2
Moderate	12
Severe	12
Severity based on MRI	
Normal	9
Stage 2	14
Stage 3	9

*DS* Doppler sonography

^a^All data are presented as frequency and percent

^b^Normal DS was considered as resistive index (RI) = 0.68–0.72; mild was considered as RI = 0.63–0.67; moderate as RI = 0.58–0.62 and severe as RI ≤ 0.57

Pearson's correlation showed that, DS of the ACA, MCA, BA all had a significant and strong correlation with MRI findings (regarding severity of asphyxia) (*r* > 0.8; *p* < 0.001). DS of the ACA and the BA had the strongest correlation with MRI findings (*r* = 0.889 and *p* < 0.001) (Table 2).

**Table 2:** Correlation between staging based on color Doppler sonography and staging based on MRI of asphyxiated neonates^a^

	DS of ACA	DS of MCA	DS of BA	MRI
DS of ACA	1	0.883**	1**	0.889**
DS of MCA	0.883**	1	0.883**	0.844**
DS of BA	1**	0.883**	1	0.889**
MRI	0.889**	0.844**	0.889**	1

*DS* Doppler sonography, *ACA* anterior cerebral artery, *MCA* middle cerebral artery, *BA* basilar artery

^a^All reported values are correlation coefficients (r)

^**^P < 0.001

In the ROC analysis, all the arteries were separately evaluated regarding their compatibility with MRI findings. DS of the ACA showed that the ideal cut-off point for diagnosing neonates with asphyxia was at a RI of ≤ 0.62 (AUC = 0.957 and 95% CI: 0.819–0.997; sensitivity = 95.65; specificity = 100; PPV = 100; NPV = 90.9 and NLR = 0.043).

Regarding the BA, the exact same results were recorded as the ACA (AUC = 0.957 and 95% CI: 0.819–0.997; sensitivity = 95.65; specificity = 100; PPV = 100; NPV = 90.9 and NLR = 0.043).

**Fig. 1:**
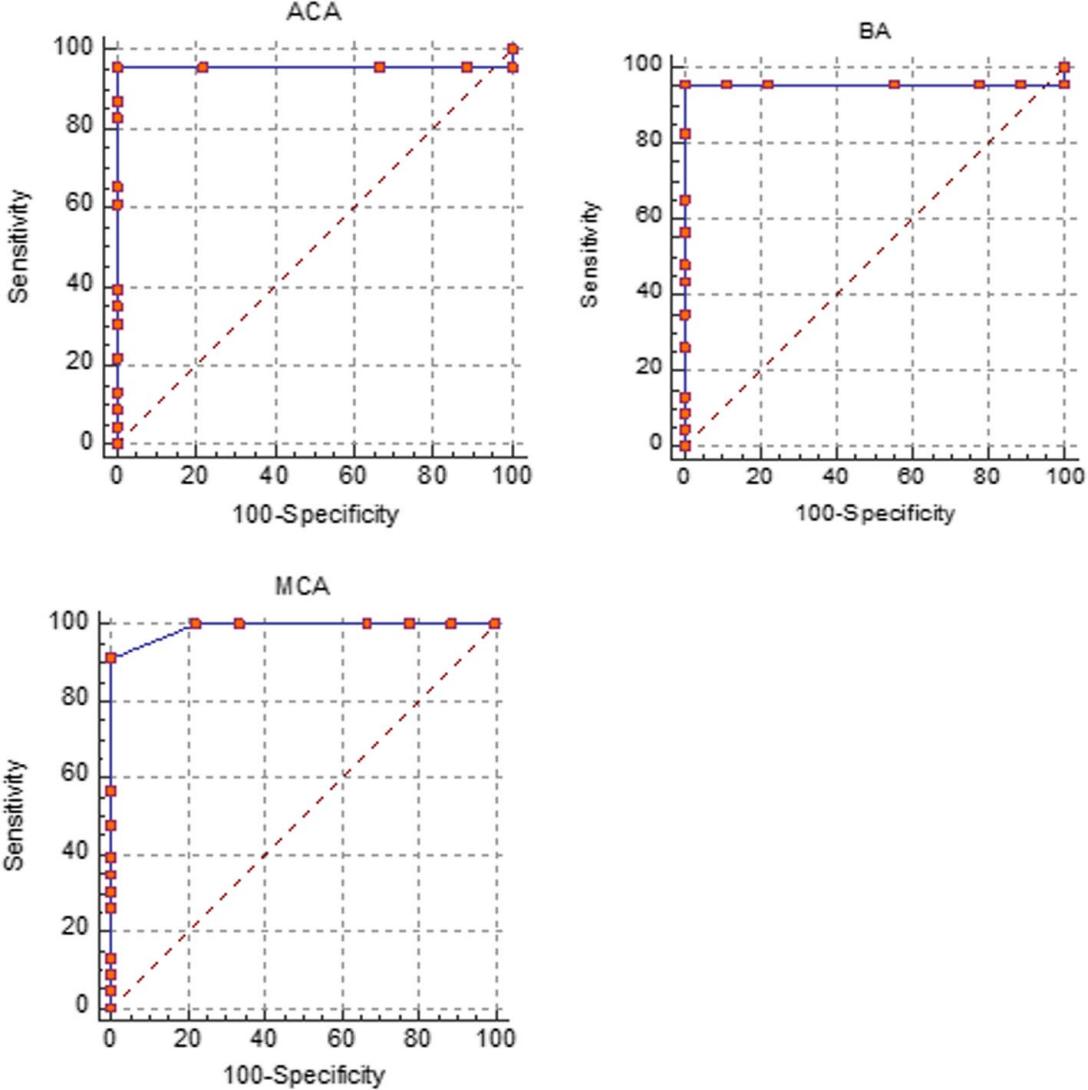
ROC curves of the ACA, MCA and BA for defining the ideal cut-off point based on resistive index for the diagnosis of perinatal asphyxia

ROC analysis of the MCA showed that, similar to the ACA and BA, a cut-off of ≤ 0.62 was the ideal cut-off point for differentiating between normal neonates and those with asphyxia (AUC = 0.990 and 95% CI: 0.873–1; sensitivity = 91.30; specificity = 100; PPV = 91.2; NPV = 100 and NLR = 0.087) (Table 3) (Fig. 1).

**Table 3:** Receiver operating characteristics analysis for the optimum cut-off point for the diagnosis of perinatal asphyxia based on resistive index in Doppler sonography^a^

	AUC	Sensitivity	Specificity	PPV	NPV	PLR	NLR
ACA							
< 0.46	-	0 (0—14.8)	100 (66.4—100)		30.3 (15.4—49)		1 (1—1)
≤ 0.62^b^	0.957 (0.819 to 0.997)	95.65 (78.1—99.9)	100 (66.4—100)	100 (84.1—100)	90.9 (58—99.8)		0.043 (0.006—0.3)
≤ 0.84	-	100 (85.2—100)	0 (0—33.6)	69.7 (51—84.6)			
MCA							
< 0.46	-	0 (0—14.8)	100 (66.4—100)		30.3 (15.4—49)		1 (1—1)
≤ 0.62^c^	0.990 (0.873 to 1)	91.30 (72.0—98.9)	100 (66.4—100)	91.2 (72.6—98.8)	100 (61.3—100)		0.087 (0.02—0.3)
≤ 0.72	-	100 (85.2—100)	0 (0—33.6)	69.7 (51—84.6)		1 (1—1)	0.96 (0.9—1)
BA							
< 0.45	-	0 (0—14.8)	100 (66.4—100)		30.3 (15.4—49)		1
≤ 0.62^d^	0.957 (0.819 to 0.997)	95.65 (78.1—99.9)	100 (66.4—100)	100 (84.1—100)	90.9 (58—99.8)		0.043 (0.006—0.3)
≤ 0.95	-	100 (85.2—100)	0 (0—33.6)	69.7 (51—84.6)		1 (1—1)	

AUC: area under curve; PPV: positive predictive value; NPV: negative predictive value; PLR: positive likelihood ratio; NLR: negative likelihood ratio; ACA: anterior cerebral artery; MCA: middle cerebral artery; BA: basilar artery

^a^All values are presented with their 95% confidence interval. Upper and lower limits were chosen on the ROC curve considering the highest sensitivity or specificity

^b^the Youden index score at this cut-off was 0.956

^c^The Youden index score at this cut-off was 0.956

^d^The Youden index score at this cut-off was 0.913

Results of pairwise comparison of ROC curves, showed that the ROC curves between the ACA and BA (which were completely similar *p* > 0.99) did not have a significant difference with that of the MCA (*p* = 0.39).

## Discussion

In this study we evaluated DS of the ACA, MCA and BA among neonates clinically diagnosed with asphyxia and compared it with MRI findings, which is the gold standard tool for the diagnosis of neonatal asphyxia, furthermore we defined a cut-off point for RI at which DS is able to detect neonatal asphyxia. We found that a RI ≤ 0.62 in DS, is the optimum cut-off point for the diagnosis of perinatal asphyxia with an accuracy of 95% (for the ACA and the BA) and 99% (for the MCA).

To the best of the author's knowledge this is the first study to compare DS findings with MRI findings in order to determine a cut-off point based on RI for the diagnosis of perinatal asphyxia. Up to this date, studies evaluating DS in asphyxia have been mostly old, have focused on prognosis (long term neuro-developmental outcome) and are less applicable in clinical practice for discriminating between neonates with asphyxia and normal neonates.

In a study by Kudreviciene et al. in 2014 [11], one year prognosis was evaluated among neonates with hypoxic brain injuries and normal neonates, using ultrasonography (US) and DS. They found that neonates with a RI ≤ 0.55 in the ACA on days 1–5 of birth, had significantly higher watershed border zone injury, thalamus, basal ganglia and cerebellar injuries.

Ilves et al. [20] also evaluated cerebral blood flow among infants with asphyxia in order to predict long term outcomes. They found that when evaluating cerebral arteries during the first 24 h of birth (similar to that of our study), infants with poor outcome or those with severe hypoxic ischemic encephalopathy had a lower RI in the BA, MCA and carotid arteries compared to a control group.

In another study [21], US indices were measured among 212 patients, in order to determine the validity of US in predicting three year adverse outcome among children with encephalopathy. In this study, US findings were evaluated during 24 and 72 h after birth. They found that among 39 neonates who had US during the first 24 h of life and had their RI measured, those with a RI < 0.56 had a 23.6 time higher chance (95% CI: 2.6–217.5, Sensitivity = 53%, specificity = 95%, PPV = 90% and NPV = 72%) of developing adverse outcomes.

A higher threshold for RI (< 0.60) in the ACA and the MCA was documented in an older study by Stark et al. (compared to similar studies) to be associated with poor five year clinical outcomes among 16 term asphyxiated neonates [22].

Some older studies have also evaluated the relationship between neurodevelopment of neonates and cerebral blood flow indices, and have mostly documented similar findings to the previously mentioned studies regarding decreased cerebral blood flow and its association with adverse long term outcomes [18, 23, 24].

In here we found that a cut-off of ≤ 0.62 for RI is a diagnostic threshold for asphyxia among full-term neonates. This is in coherence with previous literature, furthermore to date, studies that have evaluated RI cut-off points have all been based on clinical outcomes and prognosis among asphyxiated neonates and have all documented lower cut-off thresholds than that documented in our study. Perhaps a cause for the lower cut-off points documented in different studies from that documented in our study, is that not all asphyxiated children necessarily show poor long term neurodevelopmental outcomes (for example some milder forms of the disease) and so when evaluating cut-off points based on prognosis (as in the mentioned studies) a lower threshold is documented.

One of the main causes for the difference documented between studies regarding RI cut-off points, relates to the timing of the initial DS. A study documented no significant difference in RI when performing DS during specific hours of birth between children with asphyxia and normal infants, however this difference was mostly significant during the 24 h of birth between these two groups [20], thus pointing the importance of timing of DS for comparison among studies.

For facilities where MRI (as the gold standard diagnostic modality) is too expensive or is unavailable in perinatal care centers, based on our results a RI of ≤ 0.62 in DS performed on the first day of birth can be considered an appropriate diagnostic cut-off point for neonates suspected of asphyxia. This provides a tool for easy and less costly diagnosis of asphyxia and provides clinicians with an index with high precision for diagnosis of the condition.

Our study did have some limitations. Our cut-off points were based on DS performed on the first day of birth and for those neonates for whom DS is not performed on the first day, the reported cut-off points are not applicable. Another factor which is present in almost every study evaluating sonography findings, is the operator dependency and the subjective nature of sonography. This shows that for sonography to be considered an efficient tool for the evaluation of asphyxia, there is need for expert staff members to perform and evaluate sonography findings. Factors such as hypothermia and vasogenic edema are known to affect RI [25–27], considering that all our patients received appropriate ICU care and were continuously monitored none of these caused any issue or bias in our measurements. Moreover, in our center that we lack facilities such as MRI, we refer patients for MRI only after obtaining clinical stability, thus DS during the first day can be helpful for diagnosing asphyxia in centers that have limited facilities such as MRI.

## Conclusion

For evaluating neonates clinically suspected of asphyxia, especially in centers with limited facilities such as MRI, DS can be used as a first line diagnostic modality for the diagnosis of asphyxia. A RI of ≤ 0.62 is an appropriate cut-off to differentiate between normal and asphyxiated neonates with excellent accuracy.

## Data Availability

Authors and institution may request the data from the study by directly contacting the corresponding author.

## Abbreviations

DS: Doppler Sonography
ACA: Anterior Cerebral Artery
MCA: Middle Cerebral Artery
BA: Basilar Arteries
RI: Resistive Index
MRI: Magnetic Resonance Imaging
CT: Computed Tomography
PSV: Peak Systolic Velocity
EDV: End Diastolic Velocity
RI: Resistive Index
AUC: Area Under The Curve
PPV: Positive Predictive Value
NPV: Negative Predictive Value
PLR: Positive Likelihood Ratio
NLR: Negative Likelihood Ratio
